# A novel anti-human IL-1R7 antibody reduces IL-18-mediated inflammatory signaling

**DOI:** 10.1016/j.jbc.2021.100630

**Published:** 2021-04-03

**Authors:** Suzhao Li, Liqiong Jiang, Karsten Beckmann, Jesper Falkesgaard Højen, Ulrich Pessara, Nicholas E. Powers, Dennis M. de Graaf, Tania Azam, Jared Lindenberger, Elan Z. Eisenmesser, Stephan Fischer, Charles A. Dinarello

**Affiliations:** 1 Department of Medicine, University of Colorado Denver Anschutz Medical Campus, Aurora, Colorado, USA; 2 Shenzhen Eye Hospital, Shenzhen, Guangdong, China; 3 MAB Discovery GmbH, Neuried, Germany; 4 Department of Infectious Diseases, Aarhus University Hospital, Aarhus, Denmark; 5 Biophysics Core Facility, University of Colorado Denver Anschutz Medical Campus, Aurora, Colorado, USA; 6 Department of Biochemistry and Molecular Genetics, University of Colorado Denver Anschutz Medical Campus, Aurora, Colorado, USA

**Keywords:** IL-1 receptor 7 (IL-1R7), interleukin-18 (IL-18), blockade, macrophage activation syndrome (MAS), IFNγ, therapeutic, COVID-19, AOSD, adult-onset Still's disease, DSS, dextran sodium sulfate, IBD, inflammatory bowel disease, IL-18, interleukin-18, MAS, macrophage activation syndrome, PBMC, peripheral blood mononuclear cell, sHLH, secondary hemophagocytic lymphohistiocytosis, TIR, Toll-IL-1 receptor, TNSB, trinitrobenzoic sulfonic acid

## Abstract

Unchecked inflammation can result in severe diseases with high mortality, such as macrophage activation syndrome (MAS). MAS and associated cytokine storms have been observed in COVID-19 patients exhibiting systemic hyperinflammation. Interleukin-18 (IL-18), a proinflammatory cytokine belonging to the IL-1 family, is elevated in both MAS and COVID-19 patients, and its level is known to correlate with the severity of COVID-19 symptoms. IL-18 binds its specific receptor IL-1 receptor 5 (IL-1R5, also known as IL-18 receptor alpha chain), leading to the recruitment of the coreceptor, IL-1 receptor 7 (IL-1R7, also known as IL-18 receptor beta chain). This heterotrimeric complex then initiates downstream signaling, resulting in systemic and local inflammation. Here, we developed a novel humanized monoclonal anti-IL-1R7 antibody to specifically block the activity of IL-18 and its inflammatory signaling. We characterized the function of this antibody in human cell lines, in freshly obtained peripheral blood mononuclear cells (PBMCs) and in human whole blood cultures. We found that the anti-IL-1R7 antibody significantly suppressed IL-18-mediated NFκB activation, reduced IL-18-stimulated IFNγ and IL-6 production in human cell lines, and reduced IL-18-induced IFNγ, IL-6, and TNFα production in PBMCs. Moreover, the anti-IL-1R7 antibody significantly inhibited LPS- and *Candida albicans*–induced IFNγ production in PBMCs, as well as LPS-induced IFNγ production in whole blood cultures. Our data suggest that blocking IL-1R7 could represent a potential therapeutic strategy to specifically modulate IL-18 signaling and may warrant further investigation into its clinical potential for treating IL-18-mediated diseases, including MAS and COVID-19.

Initially identified as an IFNγ-inducing factor, interleukin-18 (IL-18) is a member of the IL-1 family of cytokines (1, 2, 3). Similar to IL-1β, IL-18 is synthesized as an inactive precursor requiring processing by caspase-1 into an active (mature) cytokine (4). IL-18 forms a signaling complex by binding to the IL-1 receptor 5 (IL-1R5, also known as IL-18 alpha chain), which is the ligand-binding chain for mature IL-18; however, this binding is of low affinity. In cells that express the coreceptor, termed IL-1 receptor 7 (IL-1R7, also known as IL-18 receptor beta chain), a high affinity complex is formed. With the juxtaposition of Toll-IL-1 receptor (TIR) domains in the cytosolic segment of the IL-18 receptor complex, downstream inflammatory signaling is initiated including sequential recruitment and activation of MyD88, IRAKs, TRAF6, and NFκB (1).

IL-18 is upregulated in many diseases including inflammatory bowel diseases (IBD), macrophage activation syndrome (MAS), and COVID-19 (1, 5, 6, 7, 8, 9, 10, 11, 12, 13, 14, 15, 16, 17, 18, 19, 20, 21, 22, 23, 24, 25, 26, 27, 28, 29, 30). Thus, there is considerable interest to develop IL-18 inhibitors to treat these diseases. The activity of IL-18 is kept low by its natural inhibitor, the IL-18-binding protein (IL-18BP), which provides a competing high-affinity binding site for IL-18 (31). Clinical studies reveal that blocking IL-18 with IL-18BP reduces the severe life-threatening colitis in children with the NLRC4 mutation (32). In addition, blocking IL-12, IL-18, and IFNγ has shown to reduce the severity of experimental IBD in mice (33, 34, 35). Importantly, neutralization of IL-18 with anti-IL-18 antibodies or IL-18BP is effective in both dextran sodium sulfate (DSS) and trinitrobenzoic sulfonic acid (TNSB)-induced models of IBD and reduces intestinal IFNγ and TNFα, demonstrating IL-18 as a pivotal mediator in experimental colitis (34, 36, 37). MAS, which is also known as secondary hemophagocytic lymphohistiocytosis (sHLH), is characterized by a severe hyperinflammatory state with pancytopenia, liver dysfunction, increased D-dimer and ferritin, and coagulopathy (26). A severe IL-18/IL-18BP imbalance was found in MAS patients where the plasma concentrations of IL-18 were 20–30-fold higher than in patients with rheumatic arthritis (38, 39, 40, 41, 42). In addition, MAS is observed in COVID-19 patients with severe disease (28, 43). The serum levels of IL-18 were significantly higher in the COVID-19 patients with MAS compared with COVID-19 patients without MAS (28) and were associated with disease severity and poor clinical outcome in COVID-19 patients (29, 30). Patients with a gain-of-function mutation in *NLRC4* (32) or deficiency in X-linked inhibitor of apoptosis (XIAP) (19) experience a life-threatening hyperinflammatory state with high levels of free IL-18 that is similar to MAS; treatment of these patients with IL-18BP alleviates the inflammatory state (26). In addition, markedly elevated plasma IL-18 levels are present in patients with systemic juvenile idiopathic arthritis (sJIA) or systemic inflammatory adult-onset Still's disease (AOSD), which are at high risk of developing life-threatening MAS (22, 39, 42, 44, 45). Treatment with anakinra, a natural antagonist for the IL-1 receptor, is effective for the patients with sJIA or AOSD who develop MAS (26, 46, 47). The mechanism here includes a reduction in the processing of the inactive IL-18 precursor into an active cytokine (48). Moreover, IL-18BP has also been used effectively to treat patients with refractory AOSD and sJIA and demonstrated early signs of clinical and laboratory marker efficacy (49, 50). Together, these findings suggest that IL-18 neutralization can contribute to the resolution of the hyperinflammatory state. Although IL-18 is a validated therapeutic target for treating IBD and MAS, IL-1R5 also serves as a receptor for the anti-inflammatory cytokine IL-37 (51, 52). Therefore, antibodies against IL-1R5 would concurrently block endogenous IL-37 and its anti-inflammatory functions. In addition, because of the high affinity of IL-18BP for IL-18, IL-18BP also binds IL-37 (53, 54). Thus, use of IL-18BP to reduce the activity of IL-18 has the disadvantage of binding to IL-37 and reducing the function of IL-37 in disease. In fact, several studies have reported inflammatory diseases associated with low IL-37 (55, 56, 57). The anti-inflammatory properties of IL-18BP are lost at high doses (58). Indeed, there are data revealing that blocking IL-1R5 with antibodies or using IL-18BP exacerbates inflammation (59, 60).

Different from other promiscuous accessory proteins in the IL-1 receptor family such as IL-1R3 (61), IL-1R7 is the sole accessory chain for IL-1R5 and IL-18 signaling (62). IL-1R7 is essential for the recruitment and activation of IRAK, which is required for IL-18-induced signaling and function (63, 64, 65, 66). Most importantly, anti-IL-1R7 allows for targeting IL-18 specifically without affecting endogenous IL-37 signaling and is the rationale for the development of anti-IL-1R7. The anti-IL-1R7 antibody used in the present study bound specifically to human IL-1R7 and contained the Fc-LALA mutation to prevent the triggering of FcγRs (61, 67). Using this novel antibody, we carried out *in vitro* cultures to assess the effectiveness of anti-IL-1R7 in inhibiting IL-18 activities in both human cell lines and primary cells. We found that the anti-IL-1R7 antibody specifically suppresses IL-18-mediated proinflammatory signaling and subsequent cytokine production. Data from these studies suggest that blocking IL-1R7 could be a potential therapeutic strategy to specifically modulate IL-18 signaling and IL-18-related inflammatory diseases including MAS and possibly in patients with MAS-like clinical manifestations of COVID-19.

## Results

### The binding specificity of anti-IL-1R7 antibodies to human IL-1R7

**Figure 1:**
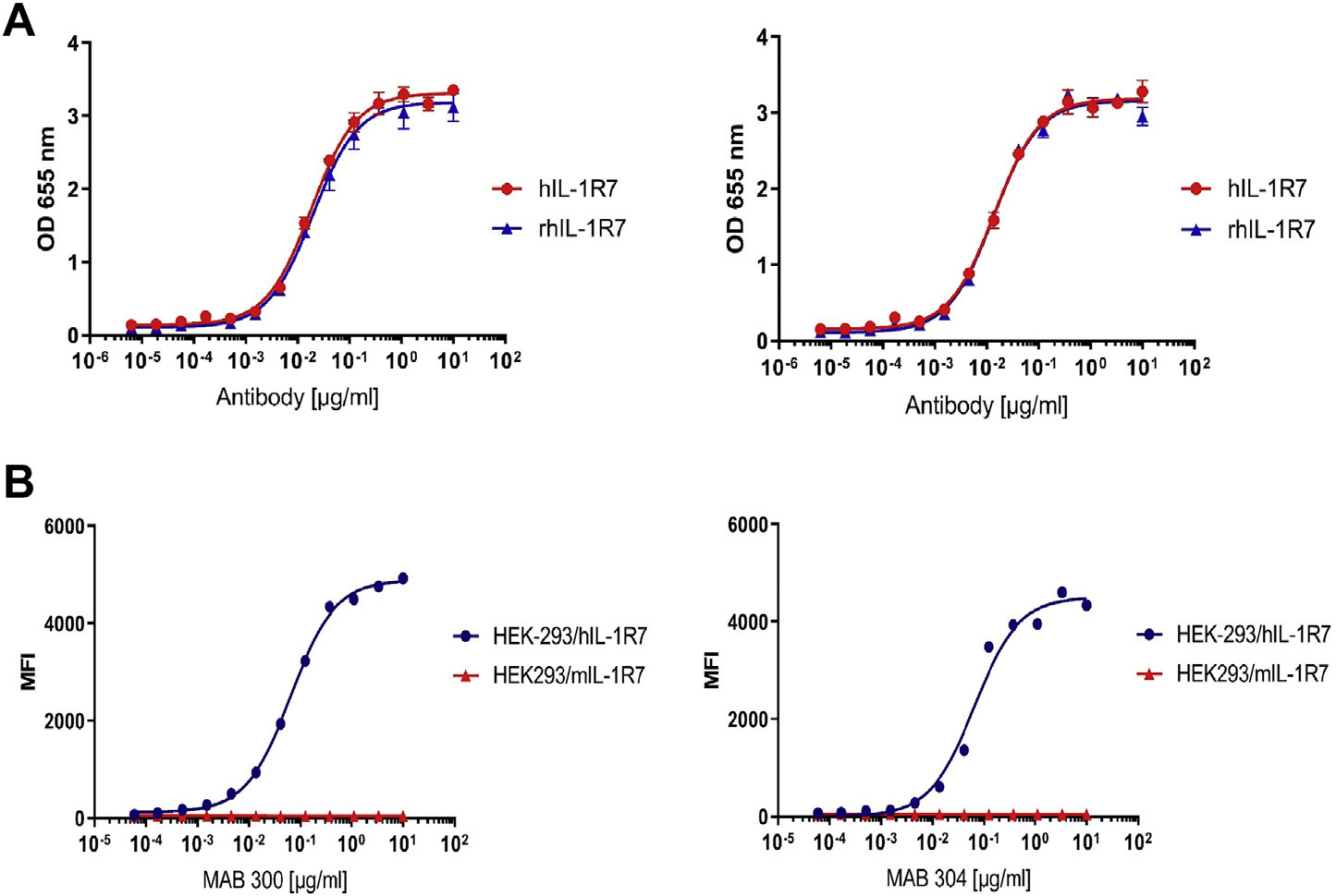
**The binding specificity of anti-IL-1R7 antibodies to human IL-1R7.***A*, dose titration curve for anti-IL-1R7 binding to immobilized recombinant human or rhesus IL-1R7 protein. ELISA data showing the *in vitro* binding affinity of anti-IL-1R7 to IL-1R7. Left curve for anti-IL-1R7 MAB300 and right curve for MAB 304. *B*, dose titration curves for anti-IL-1R7 binding to HEK-293-FreeStyle cells transiently expressing full-length human- or mouse-IL-1R7 encoding DNAs. *Left* curve for MAB300 and right curve for MAB 304.

We selected two anti-IL-1R7 antibodies to determine binding to human cell lines. These antibodies (MAB 300 and MAB 304) were humanized IgG1 and expressed with the LALA sequence. They were developed to target human IL-1R7 (hIL-1R7) and thus to inhibit assembling of the IL-18/IL-1R5/IL-1R7 ternary complex and the subsequent proinflammatory signaling of IL-18. The binding capacities of the antibodies were first tested by titration to either immobilized recombinant human IL-1R7 or recombinant rhesus monkey IL-1R7 (rhIL-1R7). As shown in Figure 1*A*, two different anti-IL-1R7 antibodies MAB300 (left) and MAB304 (right) both bind immobilized recombinant human or rhesus IL-1R7 protein dose-dependently with a maximum binding capacity achieved between the concentration of 1–10 μg/ml. The EC_50_ values of the fitted binding curves are shown in Table 1. MAB 300 binds to human and rhesus monkey IL-1R7 with similar EC_50_ values of 18.4 and 19.7 ng/ml, respectively, whereas MAB 304 binds to human and rhesus monkey IL-1R7 with EC_50_ values of 14.2 and 13.6 ng/ml, respectively. Next, we further analyzed the binding of the antibodies to cells ectopically expressing human- or mouse-IL-1R7. As presented in Figure 1*B*, similar to the recombinant protein binding in Figure 1*A*, both MAB 300 and MAB 304 antibodies bind efficiently to HEK-293-FreeStyle cells transiently expressing full-length human-IL-1R7 encoding DNA. MAB 300 binds to hIL-1R7 expressing cells in a dose-dependent manner and an EC_50_ of 64.6 ng/ml (see Table 1), while MAB 304 binds with an EC_50_ of 39.6 ng/ml. Importantly, both antibodies do not bind to mouse-IL-1R7 (mIL-1R7) expressed on HEK-293-FreeStyle cells (Fig. 1*B* and Table 1).

**Table 1:** The EC_50_ values of fitted binding curves of anti-IL-1R7 antibody to immobilized recombinant human or rhesus IL-1R7 protein (upper) or to HEK-293-FreeStyle cells transiently expressing full-length human- or mouse-IL-1R7 encoding DNAs (bottom) as shown in Figure 1

Antibody	hIL-1R7/EC_50_ (ng/ml)	rhIL-1R7/EC_50_ (ng/ml)
MAB 300	18.4	19.7
MAB 304	14.2	13.6

Antibody	HEK293/hIL-1R7 EC_50_ (ng/ml)	HEK293/mIL-1R7 EC_50_ (ng/ml)
MAB 300	64.6	n.a.
MAB 304	39.6	n.a.

EC_50_ values calculated from fitting curves in Figure 1*A* (upper) and Figure 1*B* (bottom).

### Effects of the anti-IL-1R7 antibodies on IL-18-mediated proinflammatory signaling and cytokine production in human cell lines

**Figure 2:**
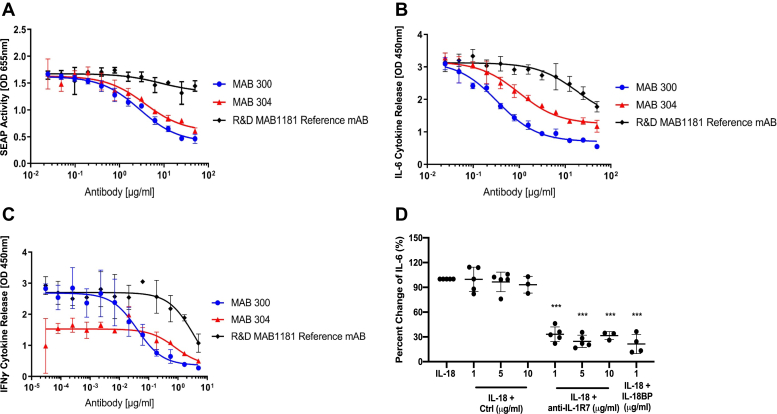
**The effect of anti-IL-1R7 antibody on IL-18-mediated proinflammatory signaling and cytokine production in different cell line cultures.***A*, inhibition of IL-18-induced gene reporter activation by anti-IL-1R7 MAB300 and MAB304 or the reference mAB MAB1181 in HEK-Blue IL-18 cells. SEAP (Secreted embryonic alkaline phosphatase) activity in the supernatant is determined as a measure of the activation of the IL-18 signaling pathway as described in Experimental procedures. *B*, inhibition of IL-18-induced IL-6 production by anti-IL-1R7 or the reference mAB in human A549-hIL-1R7/9 cells. *C*, inhibition of IL-18-induced IFNγ release by anti-IL-1R7 or the reference mAB in human KG-1 cells. *D*, effect of anti-IL-1R7 on IL-18-induced IL-6 in human A549-hIL-1R7 cell cultures. Cells were pretreated with or without isotype control antibody (Ctrl), anti-IL-1R7 or IL-18BP for at least 30 min before they were stimulated with 50 ng/ml IL-18 for 24 h. Mean ± SD Percent change of IL-18-induced IL-6 production in A549-hIL-1R7 cells treated with various concentrations of anti-IL-1R7 or its isotype control or IL-18BP (n ≧3 for all conditions). ∗∗∗*p* < 0.001 compared with IL-18 alone.

We carried out experiments using *in vitro* cell model systems to characterize the activity of the anti-hIL-1R7 antibodies in blocking IL-18-mediated proinflammatory signaling and cytokine production. First, HEK-Blue-IL-18 cells stably transfected with an NFκB-driven reporter gene construct were used to assess blockage of IL-18-induced proinflammatory signaling. Figure 2*A* shows the inhibition of IL-18-induced proinflammatory signaling by anti-IL-1R7 and the reference anti-IL-1R7 monoclonal antibody (mAB) MAB1181 in HEK-Blue IL-18 cells, respectively. Whereas the reference antibody MAB1181 reduces the production and secretion of the reporter to a limited extent, MAB300 and MAB304 significantly block IL-18-mediated signaling in this cell line with an EC_50_ value of 2851 ng/ml and 3750 ng/ml, respectively (Fig. 2*A* and Table 2 upper).

**Table 2:** The EC_50_ values of fitted binding curves of the inhibition of anti-IL-1R7 to IL-18-mediated proinflammatory signaling and cytokine production in human HEK-Blue-IL-18 cells (upper) or A549 cells stably transfected with the human IL-1R7/9 genes (middle) or KG-1 cells (bottom) as presented in Figure 2, *A*–*C*

Antibody	EC_50_ (ng/ml)
MAB 300	2851
MAB 304	3750
MAB1181 reference mAB	8315

Antibody	EC_50_ (ng/ml)
MAB300	336
MAB 304	994
MAB1181 reference mAB	>10,000

Antibody	EC_50_ (ng/ml)
MAB300	40.3
MAB 304	804
MAB1181 reference mAB	3523

EC_50_ values calculated from fitting curves in Figure 2*A* (upper), Figure 2*B* (middle), and Figure 2*C* (bottom).

We also used the human lung epithelial A549 cells stably transfected with the human IL-1R7 encoding gene alone (A549-hIL-1R7) or with both human IL-1R7 and IL-1R9 genes (A549-hIL-1R7/9), and human KG-1 cells to test the effect of anti-IL-1R7 on inhibition of IL-18-induced release of IL-6 and IFNγ. In the A549-hIL-1R7/9 cells, anti-IL-1R7 MAB300 and MAB304 significantly block IL-18-induced release of the IL-6 cytokine with an EC_50_ value of 336 and 994 ng/ml, respectively (see Fig. 2*B* and Table 2, middle). Again, the extent of the inhibition and the potency of anti-IL-1R7 are significantly higher as compared with the reference antibody MAB1181. Similarly, anti-IL-1R7 also potently inhibits IL-18-induced IFNγ release in human KG-1 cells (Fig. 2*C*). The EC_50_ value for this inhibition is 40.3 ng/ml for MAB300 and 804 ng/ml for MAB304, whereas the reference antibody MAB1181 inhibits only at very high concentrations (Fig. 2*C* and Table 2, bottom).

Overall, the results demonstrate that the newly developed anti-IL-1R7 MAB 300 and MAB 304 provide robust inhibition of IL-18-induced signaling and proinflammatory cell activation in different *in vitro* cell systems. In the systems used above, anti-IL-1R7 MAB 300 showed the best potency compared with both MAB 304 and the reference antibody, and this difference is most prominently observed in the KG-1 IFNγ release assay. Therefore, in the subsequent cell cultures, only MAB300 was further tested as an anti-IL-1R7 antibody in comparison to a human IgG1 isotype control antibody. First, we compared side by side the efficiency of anti-IL-1R7 MAB300 and isotype control antibody on IL-18-induced IL-6 in A549-hIL-1R7 cells (68). As shown in Figure 2*D*, anti-IL-1R7 robustly inhibits IL-18-induced IL-6 (∼70% reduction) in the cell culture at 1, 5, and 10 μg/ml, with similar potency as the natural IL-18 inhibitor IL-18BP. In contrast, the isotype control has no effect on IL-18-induced IL-6. We also tested the effect of anti-IL-1R7 on IL-1β-induced IL-6 and IL-1α. A moderate inhibitory effect of anti-IL-1R7 was observed on IL-1β-induced IL-6 (around 10% inhibition at 1 μg/ml and 30% at 5 μg/ml) in the same cells and no effect was observed on IL-1β-induced IL-1α (Fig. S1). Thus, we continued with our evaluation of MAB300 (now indicated as anti-IL-1R7) in primary human cell cultures.

### Effects of anti-IL-1R7 on IL-12/IL-18-induced cytokine production in human peripheral blood mononuclear cell (PBMC) cultures

**Figure 3:**
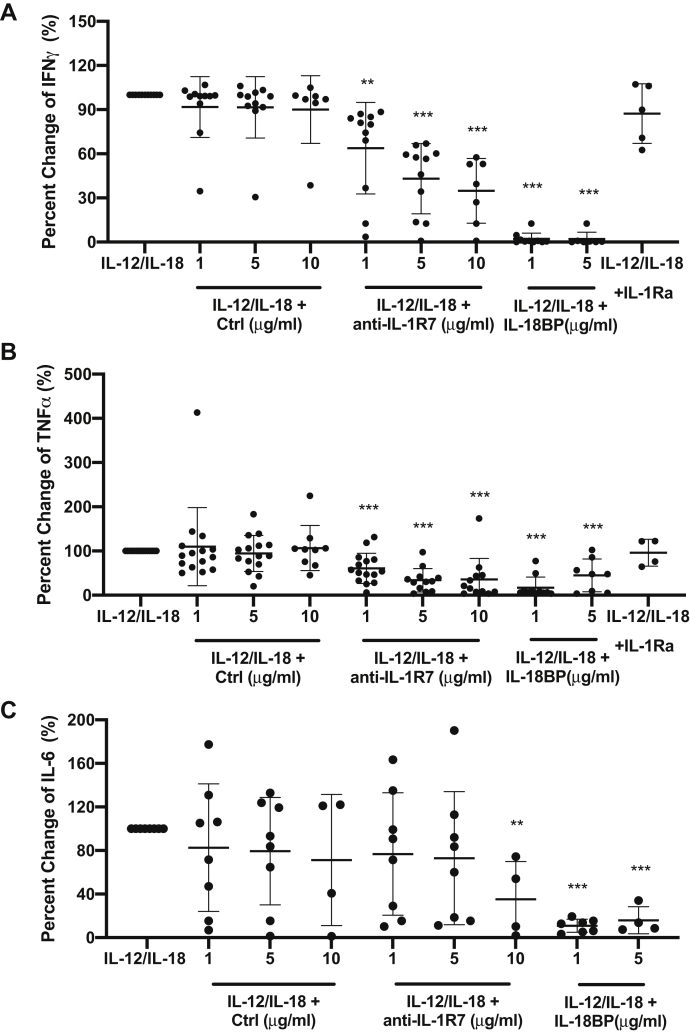
**The effect of anti-IL-1R7 antibody on IL-12/IL-18-induced cytokine production in human PBMC cultures.** Cells were pretreated with or without Ctrl, anti-IL-1R7, or IL-18BP or IL-1Ra for at least 30 min before they were stimulated with 2 ng IL-12 + 20 ng/ml IL-18 for 24 h. *A*, effect of anti-IL-1R7 on IL-12/IL-18-induced IFNγ. *B*, effect of anti-IL-1R7 on IL-12/IL-18-induced TNFα. *C*, effect of anti-IL-1R7 on IL-12/IL-18-induced IL-6. Mean ± SD Percent change of IL-12/IL-18-induced cytokine production in PBMCs with various concentrations of anti-IL-1R7 or its isotype control or IL-18BP or IL-1Ra (n ≧ 4 for all conditions). ∗∗∗*p* < 0.001, ∗∗*p* < 0.01 compared with IL-12/IL-18 alone.

We next examined the effect of anti-IL-1R7 on the production of cytokines by the combination of IL-18-plus IL-12 in primary human peripheral blood mononuclear cells (PBMCs) from healthy donors. IL-12 increases the expression of IL-1R5 and IL-1R7 and enhances the IL-18-induced IFNγ production in lymphocytes and human PBMCs (69, 70). Similar to its effect on IL-18-induced IFNγ in KG-1 cells (Fig. 2*C*), anti-IL-1R7 significantly inhibited IL-12/IL-18-induced IFNγ production in PBMCs in a dose-dependent manner, with a 65% reduction at 10 μg/ml (Fig. 3*A*). A reduction of 95% by IL-18BP was found in the same cells. In contrast, the reference antibody MAB1181 does not inhibit IL-12/IL-18-induced IFNγ significantly in PBMCs (Fig. S2). Similar to IL-18BP, anti-IL-1R7 also inhibits IL-12/IL-18-induced TNFα production (Fig. 3*B*). At 10 μg/ml, anti-IL-1R7 reduced IL-12/IL-18-induced IL-6 release in PBMCs by 65%, in comparison to a reduction of 85% by IL-18BP (Fig. 3*C*). In contrast, IL-1Ra, which is a natural antagonist for IL-1 signaling, has no effect on IL-12/IL-18-induced cytokines.

### Effects of anti-IL-1R7 antibody on LPS-induced cytokine production in human PBMC and whole blood cultures

**Figure 4:**
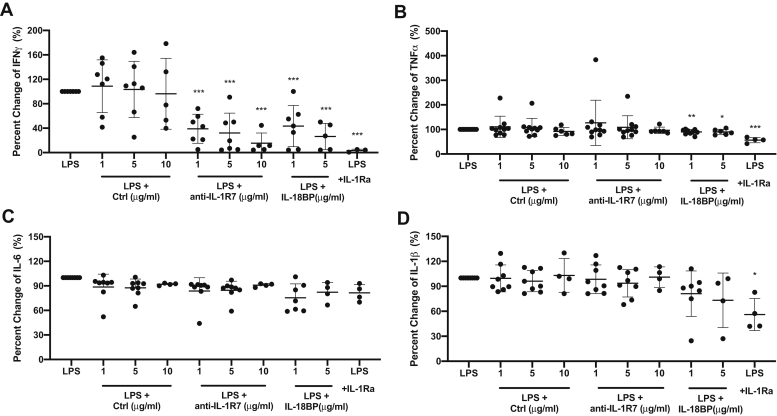
**The effect of anti-IL-1R7 antibody on LPS-induced cytokine production in human PBMC culture.** Cells were pretreated with or without Ctrl, anti-IL-1R7, or IL-18BP or IL-1Ra for at least 30 min before they were stimulated with 10 ng/ml LPS for 24 h. *A*, effect of anti-IL-1R7 on LPS-induced IFNγ. *B* Effect of anti-IL-1R7 on LPS-induced TNFα. *C*, effect of anti-IL-1R7 on LPS-induced IL-6. *D*, effect of anti-IL-1R7 on LPS-induced IL-1β. Mean ± SD Percent change of LPS-induced cytokine production in PBMCs with various concentrations of anti-IL-1R7 or its isotype control or IL-18BP or IL-1Ra (n ≧4 for all conditions). ∗∗∗*p* < 0.001, ∗∗*p* < 0.01, and ∗*p* < 0.05, compared with LPS alone.

**Figure 5:**
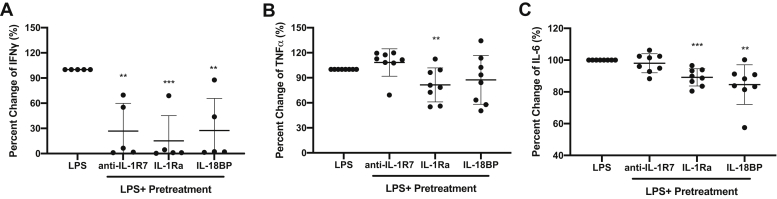
**The effect of anti-IL-1R7 antibody on LPS-induced cytokine production in human whole blood culture.** Human whole blood cultures were pretreated with or without 5 μg/ml anti-IL-1R7 or 10 μg/ml IL-1Ra or 1 μg/ml IL-18BP for at least 30 min before they were stimulated with 10 ng/ml LPS for 3 days. *A*, effect of anti-IL-1R7 on LPS-induced IFNγ in the whole blood culture. *B*, effect of anti-IL-1R7 on LPS-induced TNFα in the whole blood culture. *C*, effect of anti-IL-1R7 on LPS-induced IL-6 in the whole blood culture. Mean ± SD Percent change of LPS-induced cytokine production in human whole blood culture with various concentrations of anti-IL-1R7 or IL-18BP or IL-1Ra (n ≧ 5). ∗∗∗*p* < 0.001, ∗∗*p* < 0.01, and ∗*p* < 0.05, compared with LPS alone.

As an IFNγ-inducing factor, IL-18 is constitutively expressed in fresh human PBMCs and whole blood (71). It is required for and also facilitates LPS-induced IFNγ in PBMCs and whole blood (71, 72). Thus, we further measured the effects of anti-IL-1R7 on the production of LPS-induced IFNγ and other cytokines in both PBMC and whole blood cultures. As shown in Figure 4*A*, anti-IL-1R7 specifically inhibited 24-h LPS-induced IFNγ in PBMC cultures, with a nearly 85% inhibition at 10 μg/ml of the antibody. This reduction is comparable to or even greater than the reduction by IL-18BP. There was no significant reduction on LPS-induced TNFα, IL-6 and IL-1β using either anti-IL-1R7 or IL-18BP (Fig. 4, *B*–*D*). In contrast, while not affecting IL-12/18-induced cytokines, IL-1Ra reduced LPS-induced IFNγ, TNFα, and IL-1β, consistent with an important role of IL-1 in LPS-induced inflammatory signaling (73). Similar results were observed in 3-day LPS-induced cytokines (Fig. S3). In parallel, we also assessed the function of anti-IL-1R7 on LPS-induced cytokines in whole blood cultures. In line with the effects observed in PBMC cultures, anti-IL-1R7 inhibited LPS-induced IFNγ (∼73%) in whole blood cultures. In these same cultures, we found no reduction in LPS-induced TNFα or IL-6 (Fig. 5). IL-18BP and IL-1Ra suppressed LPS-induced IFNγ, TNFα, or IL-6, similarly as observations in PBMC cultures.

### Effects of anti-IL-1R7 antibody on *Candida*-induced cytokine production in PBMC cultures

*Candida* has been shown to markedly induce Th1 lymphocyte activation and IFNγ production in PBMCs after 48 h (74) and IL-18 mediates the *Candida*-induced IFNγ production (75). We thus assessed the effect of anti-IL-1R7 on *Candida*-induced cytokine production. As presented in Figure S4, anti-IL-1R7 reduced *Candida*-induced IFNγ by 20% significantly (Fig. S4*A*). In comparison to the isotype control antibody, there was no effect of the anti-IL-1R7 on the level of *Candida*-induced TNFα, IL-6, or IL-1β (Fig. S4, *B*–*D*). Similarly, IL-18BP significantly inhibited *Candida*-induced IFNγ by 20%, but not on other cytokines. In contrast, IL-1Ra, showing no obvious effect on *Candida*-induced IFNγ, significantly reduced *Candida*-induced TNFα (40%), IL-6 (57%), and IL-1β (60%) in the PBMC cultures (Fig. S4).

## Discussion

**Figure 6:**
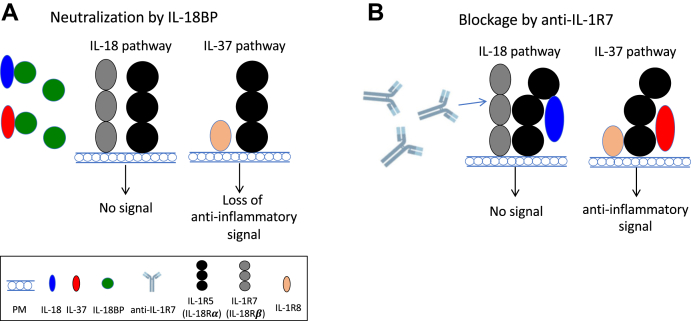
**Models depicting how IL-18BP and anti-IL-1R7 antibodies inhibit the IL-18 pathway.***A*, the model of how IL-18BP modulates the function of IL-18 or IL-37. *B*, the model of how anti-IL-1R7 regulates the function of IL-18 while leaving the IL-37 pathway intact. For simplicity, only the extracellular domains of the receptor complexes are shown.

In summary, our data have confirmed the binding affinity and specificity of the novel LALA-mutated anti-human IL-1R7 (MAB 300) to both recombinant and cell surface expressed human IL-1R7 and demonstrated the efficacy of the antibody in inhibiting IL-18-mediated inflammatory signaling, responses, and cytokine production. We observed similar trends of inhibitory effects between the newly developed anti-IL-1R7 and IL-18BP on IL-18-mediated inflammatory responses and cytokine production. However, different from IL-18BP, our antibody selectively binds the human IL-1R7 with a high affinity in the nanomolar range and prevents IL-18 signaling without affecting the anti-inflammatory signaling of IL-37 (Figure 6 and Fig. S5) (26). The antibody does not interfere with IL-1R5, which is needed for binding IL-37 (52). And it does not interfere with IL-18BP binding to IL-37 (53). In fact, any reduction in IL-37 levels due to binding to IL-18BP can result in greater inflammation. Thus, the specificity of anti-IL-1R7 for IL-18 blockade is the rationale to prevent IL-18 activity.

In any IL-18-related pathological condition, the outcome of blocking IL-18 correlates with the concentration of free, active IL-18, the surface level of IL-1R5, the presence of IL-1R7, and the level of IL-18BP (1). In health, the naturally occurring IL-18BP binds IL-18 with a high affinity (0.5 nM) and markedly low concentrations of free IL-18 are available, if any, to trigger the IL-1R5. IL-1R5 is thus available to bind the anti-inflammatory cytokine IL-37. However, in diseases with hyperinflammatory status, such as MAS, large amounts of free IL-18 are produced to bind IL-1R5 and less IL-1R5 becomes available for IL-37 to function as an anti-inflammatory cytokine. On the other hand, if the concentration of IL-18BP increases and exceeds the need to bind IL-18, IL-37 can bind to the excess IL-18BP and is not available for promoting its anti-inflammatory portfolio (52, 53, 76). This concept fits well with a recent finding from a Dutch study where 300 patients at high risk for a cardiovascular event had high levels of IL-18BP (77). In that study, biomarkers of risk such as CRP correlated with the level of IL-18BP. Therefore, considering that IL-18BP or anti-IL-1R5 antibodies could interfere the anti-inflammatory activity of IL-37 in humans (51, 52, 53, 54), the clinical application of anti-IL-1R7 would be more precise in treating IL-18-mediated diseases.

In contrast to MAB1181, a current commercially available reference monoclonal antibody for anti-IL-1R7, our novel anti-IL-1R7 (MAB300) shows a twofold greater ability in suppressing IL-18-activated NFκB signaling and IL-6 or IFNγ production and a higher efficiency than another candidate antibody we developed (MAB304) (Fig. 2). In the experiment of Figure 2*C*, we unexpectedly observed a lower basal level of secreted IFNγ for MAB304. This may be related to some variation in the number of cells seeded on the culture plate. In addition, it should be noted that the EC_50_ values for inhibition of IL-18-induced cell activation differ significantly between the different cell systems tested (Table 2). This may be explained by the artificial gene reporter setup used in HEK-Blue IL-18 cells to measure IL-18 blockade and the expression levels of transfected IL-18 receptors in the A549-IL-1R7/9 cells. In our PBMC cultures, the reference antibody MAB1181 does not significantly inhibit IL-12/IL-18-induced IFNγ production in primary PBMCs (Fig. S2). Targeting at different protein fragments in IL-1R7 as immunogens may affect the bioactivities of the antibodies robustly *in vivo.* Recently, Liu *et al* reported the development of a synthetic human antibody *via* phage-display system, which can antagonize IL-1R7 and its signaling through an allosteric mechanism (78). We compared our data from a similar KG1 assay for IL-18-induced IFNγ release and observed an IC50 of 40 ng/ml with our lead candidate (MAB300) (Fig. 2*C*). This corresponds to an IC50 of 0.26 nM and is more effective than the reported IgG 3131 with an IC50 of 3 nM by Liu *et al.* (78). Moreover, our antibodies have incorporated an Fc-LALA (L234A/L235A) substitution with an advantage to prevent the triggering of FcγRs (61, 67). In the previous study using an Fc-mediated gene reporter assay (61), antibody with the LALA mutation completely abrogated Fc-mediated effector cell functions without cytotoxic potential.

In our human A549 cell study, it is noteworthy that we tested the anti-IL-1R7 in two different but similar A549 cell lines. In the A549 cell line expressing both IL-1R7 and IL-1R9, MAB1181 was used as the reference monoclonal anti-IL-1R7 antibody to compare the inhibitory effects of anti-IL-1R7 antibodies MAB 300 and MAB304 on IL-18-induced IL-6 (Fig. 2*B*). In the A549-IL-1R7 cell line where only IL-1R7 was stably overexpressed, an anti-Digoxigenin antibody was used as a nonbinding isotype control in parallel to compare the effect of anti-IL-1R7 (MAB300) on IL-18-induced IL-6 (Fig. 2*D*). In both experiments, MAB300 suppressed IL-18-induced IL-6 with similar reductions: ∼66% inhibition at 1 μg/ml and ∼70% inhibition at 10 μg/ml. The results from the two A549 cell line cultures confirmed the dependency of IL-1R7 in IL-18 signaling. Not surprisingly, the expression of IL-1R9 had no effect on the activity of IL-18. In contrast to the marked inhibition on IL-18-induced IL-6 production from A549-IL-1R7 cells by anti-IL-1R7, we detected a moderate reduction on IL-1β-induced IL-6. No effect was observed on IL-1β-induced IL-1α production (Fig. S1). As there are no T cells or NK cells in the A549 cell cultures, there is likely no role of IFNγ on the IL-1β signaling as can take place in PBMC cultures (79). The minor inhibition on IL-1β-induced IL-6 we observed in the A549 cells may be explained by an effect of the anti-IL-1R7 on the NF-κB signaling in the cells *via* the stably overexpressed IL-1R7 (68).

We further characterized the function of the anti-IL-1R7 antibody on primary human PBMC and whole blood cultures. First, we assessed the effect of the antibody on IL-12/IL-18-induced IFNγ secretion in PBMCs. This is a direct IL-18-stimulated inflammatory response and the suppressive effect of anti-IL-1R7 is straightforward and dose-dependent. Interestingly, besides IFNγ, both anti-IL-1R7 and IL-18BP inhibit IL-12/IL-18-induced TNFα production (Fig. 3*B*). This is consistent with previous studies where IL-18 was shown to induce TNFα production and IL-18BP reduces *Staphylococcus epidermidis*–induced TNF-α production in human whole blood (80, 81, 82, 83). Though IL-12/IL-18-induced IL-1β production was below detection, an inhibitory effect was observed on IL-12/IL-18-induced intracellular IL-1α by anti-IL-1R7 and IL-18BP (Fig. S6*A*), indicating a potential impact of IL-18 signaling on IL-1. It is not surprising that in comparison to IL-18BP, our anti-IL-1R7 presented a relatively weaker inhibition on IL-12/IL-18-induced cytokine production (Fig. 3). IL-18BP is known to be a natural inhibitor to IL-18 (31) and directly binds and blocks the activity of IL-18. In contrast, anti-IL-1R7 indirectly suppresses the activity of IL-18 by blocking the function of its coreceptor IL-1R7. The detailed mechanism by which our novel anti-IL-1R7 suppresses the function of endogenous IL-1R7 and how it regulates the association of IL-1R7 to IL-1R5 and/or IL-18 to initiate the downstream IL-18 signaling would be worthy of further investigation.

In the attempt to test the effects of anti-IL-1R7 on pathogen-activated inflammatory responses where other signaling besides IL-18 is involved, we found that the antibody also significantly suppressed LPS-induced IFNγ in both PBMC and whole blood cultures (Figs. 4 and 5; and Fig. S3). This is consistent with the requirement of IL-18 in LPS-induced IFNγ production in PBMCs (71, 84). Though no obvious effect was observed in LPS-induced TNFα, IL-6, or IL-1β, LPS-induced intracellular IL-1α was found to be downregulated by anti-IL-1R7 (Fig. S6*B*), suggesting a potential involvement of IL-18 on LPS-mediated IL-1 signaling. In the *Candida* model, a significant suppression was detected in *Candida*-induced IFNγ while no effect was observed on other cytokines such as TNFα, IL-6, IL-1β, or IL-1α (Figs. S4 and S6*C*). Moreover, the effect of either anti-IL-1R7 or IL-18BP on *Candida*-induced IFNγ production was smaller than that of LPS and IL-12/IL-18. We postulate that this might be due to the various pattern recognition receptors and signaling pathways that mediate the complexed *Candida*-host immune responses (85, 86), in which IL-18 plays a relatively minor role.

Notably, in the recent COVID-19 outbreak, a cytokine profile resembling sHLH was found to be associated with COVID-19 disease severity, characterized by increased cytokines such as IFNγ, MCP-1, MIP1-α, and TNFα (87). Moreover, high levels of IL-18 were found to be associated with disease severity and poor clinical outcome in COVID-19 patients (27, 29, 30, 88, 89). These findings shed light on the role of IL-18 in the COVID-19 pathogenesis and indicate a potential of high plasma IL-18 as a disease marker in the prognosis and treatment of severe COVID-19 patients. Importantly, the COVID-19 pandemic has brought attention to a virally induced hyperinflammatory lung injury, sometimes evolving to cytokine storm syndrome, multiple-organ failure, and death (90, 91). This finding mirrors what has been observed in MAS (92, 93). Indeed, MAS was found to present in some COVID-19 patients and a significantly higher serum IL-18 level was observed in the patients with MAS than patients without MAS (28, 43). In the same study, patients with or without MAS also present higher serum IL-18 than healthy controls and IL-18 level was significantly higher in nonsurvivors compared with survivors (28). Similarly, in SARS caused by SARS-CoV-1, IL-18 concentration was found to be considerably elevated compared with those in healthy subjects and was significantly higher in nonsurvivors compared with survivors (94, 95). IL-18 was involved in an IFNγ-related cytokine storm in the patients (94). Moreover, IL-18 and IL-1R7 are found to be highly expressed in cell-to-cell communication among immune cells in COVID-19 patients (96) and elevated IFNγ was observed in COVID-19 patients in line with increased IL-18 levels (30, 88, 89). However, their exact function remains unknown.

Altogether, results from this study set the stage for future studies to characterize the *in vivo* function of this novel antibody in clinical studies of IL-18-mediated diseases such as MAS, IBD, and rheumatic diseases (5, 26). Further research on its application will not only provide new mechanistic insights into the function of IL-18 in disease, but also will likely identify novel therapeutic targets for treating IL-18-mediated diseases. In particular, patients carrying the NLRC4 mutation with life-threatening enterocolitis could potentially benefit from such an antibody specific to IL-18 inhibition (32). Whether anti-IL-1R7 antibody could also help to reduce the cytokine storm and associated organ damages in COVID-19 will also be worthy of further exploration.

## Experimental procedures

### Antibodies and reagents

The anti-human IL-1R7 antibody was generated by immunization of New Zealand white rabbits (Charles River Laboratories) with human recombinant IL-1R7 protein. Anti-human IL-1R7 antibody and nonbinding isotype control antibody were produced as hIgG1-LALA isotype in HEK293-FreeStyle cells from Thermo Fisher Scientific and purified from the supernatant using protein-A affinity chromatography followed by size-exclusion chromatography (MAB Discovery GmbH). The antibodies have an incorporated double substitution, LALA that significantly reduces binding to FcγRs and thus can avoid Fc-mediated effector functions (61, 67). The antibodies were then dissolved in the buffer with 20 mM Histidine, 140 mM NaCl at pH 6, divided into aliquots, and stored at –80 °C before use. Lipopolysaccharide (LPS) *Escherichia coli* (055:B5) was purchased from Sigma. Heat-killed *Candida albicans* UC820 was kindly provided by Professor Mihai Netea (Radboud University Medical Centre). Human IL-12 was from PeproTech. Human IL-18 and IL-1β were from Bio-Techne. Recombinant human IL-37 46-218 was produced as described earlier (97). The reference anti-IL-1R7 monoclonal antibody MAB1181 of R&D Systems (R&D MAB1181 Reference mAB) and the anti-IL-37 monoclonal antibody were from Bio-Techne. Clinical-grade recombinant human IL-18BP was a gift provided by Serono pharmaceutical research institute (SPRI). Human IL-1Ra (Anakinra) was the kind gift of Amgen. For cytokine measurements, the corresponding ELISA DuoSets kits for human IL-1β, TNFα, IL-6, IFNγ, and IL-1α were from Bio-Techne.

### Immobilized ELISA binding of anti-IL-1R7 to human IL-1R7

Nunc 384-well MaxiSorp plates were coated with recombinant human IL-1R7 extracellular domain (hIL-1R7-FC; MAB Discovery GmbH), or recombinant rhesus monkey IL-1R7 extracellular domain (Sino Biological Inc; #90122-C08H), at a concentration of 0.5 μg/ml in PBS for 60 min at room temperature. Plates were washed three times with wash buffer (PBS 0.1% Tween) and blocked with PBS, 2% BSA, 0.05% Tween for 60 min at room temperature. After three washes with wash buffer, antibodies were added in ELISA buffer (PBS, 0.5% BSA, 0.05% Tween) at concentrations ranging from 10 μg/ml to 0.006 ng/ml (1:3 dilution series) and were incubated for 60 min at room temperature. Plates were washed three times with wash buffer, followed by incubation with anti-human-F(ab)_2,_ peroxidase-linked secondary antibody (goat, AbD Serotec) at a dilution of 1:5000 in ELISA buffer for 60 min at room temperature. Plates were washed six times with wash buffer before TMB substrate solution (Invitrogen; 15 μl/well) was added. After 5 min of incubation, stop solution (1 M HCl, 15 μl/well) was added and absorbance (450 nm/620 nm) measured using a Tecan M1000 plate reader. Fitting curves and EC_50_ calculation were done using GraphPad Prism 8.

### Cell binding of anti-IL-1R7 to human IL-1R7

HEK-293-FreeStyle cells were transfected with DNAs encoding full-length human or mouse-IL-1R7 and using 293-Free Transfection Reagent (Merck). Twenty-four hours after transfection, cells were seeded in a 96-well round bottom plate at a cell density of 1 × 10^6^ cells/ml in stain buffer (BD). Anti-hIL-1R7 antibody was added to a final concentration ranging from 10 μg/ml to 0.06 ng/ml and incubated for 1 h in the dark at 4 °C. Cells were washed once with 150 μl DPBS and incubated with Alexa Fluor 488-conjugated goat F(ab)2 anti-human IgG (H + L) (Jackson ImmunoResearch Laboratories; Cat. no. 109-546-003) at a concentration of 0.8 μg/ml in stain buffer. Cells were washed once with 150 μl DPBS and resuspended in 150 μl stain buffer containing 1:500 diluted DRAQ7 solution (Abcam; Cat: ab109202; 0.3 mM). Cells were analyzed using a BD FACSVerse flow cytometer.

### PBMC cultures

The study was approved by (COMIRB) Colorado Medical Institutional Review Board and abides by the Declaration of Helsinki principles. Venous blood from healthy consenting donors was drawn into lithium heparin containing tubes and PBMCs were isolated using centrifugation over Ficoll-Hypaque cushions as previously described (51, 98, 99). Cells were washed three times with saline and resuspended in RPMI at 5 × 10^6^/ml. For IL-12/IL-18 stimulation, 0.5 × 10^6^ cells were seeded per well in 96-round bottom well plates and cultured in a total of 200 μl for 24 h, with or without the combination of 2 ng/ml IL-12 + 20 ng/ml IL-18 in the presence of different concentrations of control antibody or the reference antibody MAB1181, anti-IL-1R7, or IL-18BP and IL-1Ra. Aliquots of the control antibody or anti-human IL-1R7 or IL-18BP and IL-1Ra were freshly diluted in warm RPMI to different concentrations for experiments. For LPS stimulation, 0.5 × 10^6^ cells were seeded per well in 96-flat bottom well plates and cultured in a total of 200 μl for 24 h or 200 μl RPMI with 10% FBS for 3 days, with or without 10 ng/ml LPS in the presence of different concentrations of control antibody or anti-IL-1R7 antibody, or IL-18BP or IL-1Ra. For recombinant IL-37 46-218 treated PBMC experiments, IL-37 46-218 were preincubated with either Blank (RPMI medium), or 1 μg/ml anti-IL-37 monoclonal antibody, or 1 μg/ml anti-IL-1R7 antibody for at least 10 min before they were added to the cells for a 1-h pretreatment. After that, the cells were stimulated with 10 ng/ml LPS for 24 h. For cultures with heat-killed *Candida albcans*, 0.5 × 10^6^ cells were seeded per well in 96-well round-bottom plate and cultured in a total of 200 μl RPMI with 10% FBS for 5 days, with or without *Candida* (10^6^ colonies per ml) (100, 101) in the presence of different concentrations of control antibody or anti-IL-1R7 antibody, IL-18BP or IL-1Ra. The antibodies or IL-18BP or IL-1Ra were added 30 min before the stimuli. After incubation times were completed, supernatants were collected by centrifugation at 400*g* for 5 min and stored at –80 °C. Cells remaining in the wells were lysed in 100 μl 0.5% triton-X in water and stored at –80 °C for intracellular IL-1α analysis.

### Human whole blood culture

One milliliter of heparinized blood was added to 12 × 75 mm round-bottom polypropylene tubes (Falcon) as described as previously (72) and then 1 ml of RPMI with or without 10 ng/ml LPS was added for stimulation in the presence of different concentrations of anti-IL-1R7 or IL-18BP or IL-1Ra. The antibody or IL-18BP or IL-1Ra was added 30 min before the stimuli. The tubes were closed tightly with the caps and were mixed by inversion. Blood was incubated upright in the sealed tubes at 37 °C for 3 days. After incubation at 37 °C, the tubes were inverted several times to mix the formed elements, and Triton X-100 was added (5%; Bio-Rad Laboratories) to a final concentration of 1%. The tubes were again inverted several times until the blood was clarified. The lysed blood was frozen at –70 °C until assay.

### HEK-Blue-IL-18 assay

HEK-Blue IL-18 cells (InvivoGen) were cultivated in DMEM, 10% FCS, and seeded out in 384-well clear, flat bottom, cell culture treated microplates (Corning) at a cell density of 12,500 cells/well in 15 μl medium. Various concentrations of anti-hIL-1R7 MAB antibodies were added in a volume of 5 μl medium and plates were incubated for 60 min at 37 °C/5% CO_2_. Recombinant human IL-18 (MBL Co Ltd) protein was added in 5 μl medium to a final concentration of 100 pg/ml and plates were incubated over night at 37 °C/5% CO_2_. In total, 5 μl cell supernatants were transferred to clear, flat-bottom polystyrene NBS microplates (Corning) containing 20 μl 2xQUANTI-Blue reagent (InvivoGen). Plates were incubated at 37 °C for 45 min and optical density was measured at 655 nm using a Tecan M1000 plate reader. Fitting curves and EC_50_ calculation were done using GraphPad Prism 8.

### A549-hIL-1R7/9 assay

Human lung epithelial A549 cells stably transfected with hIL-1R7 and hIL-1R9 encoding genes were cultured in Ham's F-12K medium containing 10% FCS. In total, 12,500 cells per well were seeded into a 384-well clear, flat-bottom, cell culture treated microplate (Corning) in 15 μl medium. After 24 h at 37 °C, 5% CO_2_, medium was removed and cells were washed three times with 25 μl 1× PBS, 0.05% Tween and then resuspended in 15 μl growth medium. Antibodies were added at different concentration in a volume of 5 μl and incubated with the cells for 60 min. hIL-18 recombinant protein (MBL Co Ltd) was then added to a final concentration of 10 ng/ml in a volume of 5 μl. Cells were incubated for 6 h at 37 °C/5% CO_2_. The human IL-6 cytokine concentration in the cell supernatant was determined using the DuoSet ELISA (Bio-Techne) according to the manufacturer's instructions. Fitting curves and EC_50_ calculation were done using GraphPad Prism 8.

### KG-1 IFNγ release assay

KG-1 cells were cultured in RPMI 1640 medium containing 20% FCS and 2 mM L-glutamine. In total, 13500 KG-1 cells per well were seeded into a Corning 384 Well Clear Flat Bottom Polystyrene NBS Microplate in a volume of 15 μl. Antibodies were added at different concentration in a volume of 7.5 μl medium and incubated with the cells for 60 min at 37 °C, 5% CO_2_. hIL-18 and TNFα recombinant protein (Bio-Techne) were then added in a volume of 7.5 μl at a final concentration of 5 ng/ml and 10 ng/ml, respectively. Cells were incubated for 48 h at 37 °C, 5% CO_2_. Human IFNγ concentrations in the cell supernatant were determined using the DuoSet Human IFNγ ELISA kit (Bio-Techne) according to the manufacturer's instructions. Fitting curves and EC_50_ calculation are done using GraphPad Prism 8.

### A549-hIL-1R7 cell culture

Human A549 cells stably overexpressing IL-1R7 or IL-18 receptor β chain were cultured in F12-K culture medium (Cellgro) supplemented with 10% FBS as described before (68). In total, 50,000 cells were seeded in 96-well flat-bottom cell culture plate and pretreated with or without different concentrations of anti-IL-1R7 or IL-18BP for 30 min. The cells were further stimulated with 50 ng/ml IL-18 or 1 ng/ml IL-1β for overnight before the supernatant was collected for IL-6 measurement. Cells remaining in the wells were lysed in 100 μl 0.5% Triton-X in water for intracellular IL-1α measurement.

### Statistical analysis

Significance of differences was evaluated with Student's two-tail *t*-test. The IL-18, IL-12/IL-18, LPS, or other inflammatory stimulus-induced cytokine production in cells without pretreatment was set at 100% unless specified. The mean percent change for each condition was calculated for each group. The data shown represent the mean ± SD.

## Data availability

All data are contained within the article and available upon request.

## Supporting information

This article contains supporting information.

## Conflict of interest

K. B. and U. P. were employed by MAB Discovery GmbH, Neuried, Germany. S. F. is the CEO of MAB Discovery GmbH. J. F. H. has received consulting fees from MAB Discovery GmbH. Other authors declare that they have no conflicts of interest with the contents of this article.

## References

[1] Dinarello C.A., Novick D., Kim S., Kaplanski G. (2013). Interleukin-18 and IL-18 binding protein. Front. Immunol..

[2] Okamura H., Tsutsui H., Komatsu T., Yutsudo M., Hakura A., Tanimoto T., Torigoe K., Okura T., Nukada Y., Hattori K., Akita K., Namba M., Tanabe F., Konishi K., Fukuda S. (1995). Cloning of a new cytokine that induces interferon-g. Nature.

[3] Dinarello C.A. (2000). Interleukin-18, a proinflammatory cytokine. Eur. Cytokine Netw..

[4] Ghayur T., Banerjee S., Hugunin M., Butler D., Herzog L., Carter A., Quintal L., Sekut L., Talanian R., Paskind M., Wong W., Kamen R., Tracey D., Allen H. (1997). Caspase-1 processes IFN-gamma-inducing factor and regulates LPS-induced IFN-gamma production. Nature.

[5] Kaplanski G. (2018). Interleukin-18: Biological properties and role in disease pathogenesis. Immunol. Rev..

[6] Pizarro T.T., Michie M.H., Bentz M., Woraratanadharm J., Smith M.F., Foley E., Moskaluk C.A., Bickston S.J., Cominelli F. (1999). IL-18, a novel immunoregulatory cytokine, is up-regulated in Crohn's disease: Expression and localization in intestinal mucosal cells. J. Immunol..

[7] Leach S.T., Messina I., Lemberg D.A., Novick D., Rubenstein M., Day A.S. (2008). Local and systemic interleukin-18 and interleukin-18-binding protein in children with inflammatory bowel disease. Inflamm. Bowel Dis..

[8] Naftali T., Novick D., Gabay G., Rubinstein M., Novis B. (2007). Interleukin-18 and its binding protein in patients with inflammatory bowel disease during remission and exacerbation. Isr. Med. Assoc. J..

[9] Monteleone G., Trapasso F., Parrello T., Biancone L., Stella A., Iuliano R., Luzza F., Fusco A., Pallone F. (1999). Bioactive IL-18 expression is up-regulated in Crohn's disease. J. Immunol..

[10] Hung J., McQuillan B.M., Chapman C.M., Thompson P.L., Beilby J.P. (2005). Elevated interleukin-18 levels are associated with the metabolic syndrome independent of obesity and insulin resistance. Arterioscler. Thromb. Vasc. Biol..

[11] Zirlik A., Abdullah S.M., Gerdes N., MacFarlane L., Schonbeck U., Khera A., McGuire D.K., Vega G.L., Grundy S., Libby P., de Lemos J.A. (2007). Interleukin-18, the metabolic syndrome, and subclinical atherosclerosis: Results from the Dallas Heart study. Arterioscler. Thromb. Vasc. Biol..

[12] Fischer C.P., Perstrup L.B., Berntsen A., Eskildsen P., Pedersen B.K. (2005). Elevated plasma interleukin-18 is a marker of insulin-resistance in type 2 diabetic and non-diabetic humans. Clin. Immunol..

[13] Hulthe J., McPheat W., Samnegard A., Tornvall P., Hamsten A., Eriksson P. (2006). Plasma interleukin (IL)-18 concentrations is elevated in patients with previous myocardial infarction and related to severity of coronary atherosclerosis independently of C-reactive protein and IL-6. Atherosclerosis.

[14] Blankenberg S., Tiret L., Bickel C., Peetz D., Cambien F., Meyer J., Rupprecht H.J., AtheroGene I. (2002). Interleukin-18 is a strong predictor of cardiovascular death in stable and unstable angina. Circulation.

[15] Mallat Z., Corbaz A., Scoazec A., Besnard S., Leseche G., Chvatchko Y., Tedgui A. (2001). Expression of interleukin-18 in human atherosclerotic plaques and relation to plaque instability. Circulation.

[16] Sawada M., Kawayama T., Imaoka H., Sakazaki Y., Oda H., Takenaka S., Kaku Y., Azuma K., Tajiri M., Edakuni N., Okamoto M., Kato S., Hoshino T. (2013). IL-18 induces airway hyperresponsiveness and pulmonary inflammation via CD4+ T cell and IL-13. PLoS One.

[17] Kang M.J., Homer R.J., Gallo A., Lee C.G., Crothers K.A., Cho S.J., Rochester C., Cain H., Chupp G., Yoon H.J., Elias J.A. (2007). IL-18 is induced and IL-18 receptor alpha plays a critical role in the pathogenesis of cigarette smoke-induced pulmonary emphysema and inflammation. J. Immunol..

[18] Novick D., Schwartsburd B., Pinkus R., Suissa D., Belzer I., Sthoeger Z., Keane W.F., Chvatchko Y., Kim S.H., Fantuzzi G., Dinarello C.A., Rubinstein M. (2001). A novel IL-18BP ELISA shows elevated serum il-18BP in sepsis and extensive decrease of free IL-18. Cytokine.

[19] Wada T., Kanegane H., Ohta K., Katoh F., Imamura T., Nakazawa Y., Miyashita R., Hara J., Hamamoto K., Yang X., Filipovich A.H., Marsh R.A., Yachie A. (2014). Sustained elevation of serum interleukin-18 and its association with hemophagocytic lymphohistiocytosis in XIAP deficiency. Cytokine.

[20] Put K., Avau A., Brisse E., Mitera T., Put S., Proost P., Bader-Meunier B., Westhovens R., Van den Eynde B.J., Orabona C., Fallarino F., De Somer L., Tousseyn T., Quartier P., Wouters C. (2015). Cytokines in systemic juvenile idiopathic arthritis and haemophagocytic lymphohistiocytosis: Tipping the balance between interleukin-18 and interferon-gamma. Rheumatology (Oxford).

[21] Maeno N., Takei S., Imanaka H., Yamamoto K., Kuriwaki K., Kawano Y., Oda H. (2004). Increased interleukin-18 expression in bone marrow of a patient with systemic juvenile idiopathic arthritis and unrecognized macrophage-activation syndrome. Arthritis Rheum..

[22] Shimizu M., Nakagishi Y., Inoue N., Mizuta M., Ko G., Saikawa Y., Kubota T., Yamasaki Y., Takei S., Yachie A. (2015). Interleukin-18 for predicting the development of macrophage activation syndrome in systemic juvenile idiopathic arthritis. Clin. Immunol..

[23] Ojala J.O., Sutinen E.M. (2017). The role of interleukin-18, oxidative stress and metabolic syndrome in Alzheimer's disease. J. Clin. Med..

[24] Dinarello C.A. (2007). Interleukin-18 and the pathogenesis of inflammatory diseases. Semin. Nephrol..

[25] Mavragani C.P., Spyridakis E.G., Koutsilieris M. (2012). Adult-Onset Still's disease: From pathophysiology to targeted therapies. Int. J. Inflam.

[26] Dinarello C.A. (2019). The IL-1 family of cytokines and receptors in rheumatic diseases. Nat. Rev. Rheumatol..

[27] Satis H., Ozger H.S., Aysert Yildiz P., Hizel K., Gulbahar O., Erbas G., Aygencel G., Guzel Tunccan O., Ozturk M.A., Dizbay M., Tufan A. (2020). Prognostic value of interleukin-18 and its association with other inflammatory markers and disease severity in COVID-19. Cytokine.

[28] Kerget B., Kerget F., Aksakal A., Askin S., Saglam L., Akgun M. (2021). Evaluation of alpha defensin, IL-1 receptor antagonist, and IL-18 levels in COVID-19 patients with macrophage activation syndrome and acute respiratory distress syndrome. J. Med. Virol..

[29] Rodrigues T.S., de Sa K.S.G., Ishimoto A.Y., Becerra A., Oliveira S., Almeida L., Goncalves A.V., Perucello D.B., Andrade W.A., Castro R., Veras F.P., Toller-Kawahisa J.E., Nascimento D.C., de Lima M.H.F., Silva C.M.S. (2021). Inflammasomes are activated in response to SARS-CoV-2 infection and are associated with COVID-19 severity in patients. J. Exp. Med..

[30] Flament H., Rouland M., Beaudoin L., Toubal A., Bertrand L., Lebourgeois S., Rousseau C., Soulard P., Gouda Z., Cagninacci L., Monteiro A.C., Hurtado-Nedelec M., Luce S., Bailly K., Andrieu M. (2021). Outcome of SARS-CoV-2 infection is linked to MAIT cell activation and cytotoxicity. Nat. Immunol..

[31] Novick D., Kim S.-H., Fantuzzi G., Reznikov L., Dinarello C.A., Rubinstein M. (1999). Interleukin-18 binding protein: A novel modulator of the Th1 cytokine response. Immunity.

[32] Canna S.W., Girard C., Malle L., de Jesus A., Romberg N., Kelsen J., Surrey L.F., Russo P., Sleight A., Schiffrin E., Gabay C., Goldbach-Mansky R., Behrens E.M. (2017). Life-threatening NLRC4-associated hyperinflammation successfully treated with IL-18 inhibition. J. Allergy Clin. Immunol..

[33] Neurath M.F., Fuss I., Kelsall B.L., Stuber E., Strober W. (1995). Antibodies to interleukin 12 abrogate established experimental colitis in mice. J. Exp. Med..

[34] Siegmund B., Fantuzzi G., Rieder F., Gamboni-Robertson F., Lehr H.A., Hartmann G., Dinarello C.A., Endres S., Eigler A. (2001). Neutralization of interleukin-18 reduces severity in murine colitis and intestinal IFN-g and TNF-a production. Am. J. Physiol. Regul. Integr. Comp. Physiol..

[35] Ito R., Shin-Ya M., Kishida T., Urano A., Takada R., Sakagami J., Imanishi J., Kita M., Ueda Y., Iwakura Y., Kataoka K., Okanoue T., Mazda O. (2006). Interferon-gamma is causatively involved in experimental inflammatory bowel disease in mice. Clin. Exp. Immunol..

[36] Ten Hove T., Corbaz A., Amitai H., Aloni S., Belzer I., Graber P., Drillenburg P., van Deventer S.J., Chvatchko Y., Te Velde A.A. (2001). Blockade of endogenous IL-18 ameliorates TNBS-induced colitis by decreasing local TNF-alpha production in mice. Gastroenterology.

[37] Sivakumar P.V., Westrich G.M., Kanaly S., Garka K., Born T.L., Derry J.M., Viney J.L. (2002). Interleukin 18 is a primary mediator of the inflammation associated with dextran sulphate sodium induced colitis: Blocking interleukin 18 attenuates intestinal damage. Gut.

[38] Mazodier K., Marin V., Novick D., Farnarier C., Robitail S., Schleinitz N., Veit V., Paul P., Rubinstein M., Dinarello C.A., Harle J.R., Kaplanski G. (2005). Severe imbalance of IL-18/IL-18BP in patients with secondary hemophagocytic syndrome. Blood.

[39] Girard C., Rech J., Brown M., Allali D., Roux-Lombard P., Spertini F., Schiffrin E.J., Schett G., Manger B., Bas S., Del Val G., Gabay C. (2016). Elevated serum levels of free interleukin-18 in adult-onset Still's disease. Rheumatology (Oxford).

[40] Weiss E.S., Girard-Guyonvarc'h C., Holzinger D., de Jesus A.A., Tariq Z., Picarsic J., Schiffrin E.J., Foell D., Grom A.A., Ammann S., Ehl S., Hoshino T., Goldbach-Mansky R., Gabay C., Canna S.W. (2018). Interleukin-18 diagnostically distinguishes and pathogenically promotes human and murine macrophage activation syndrome. Blood.

[41] Gao Z., Wang Y., Wang J., Zhang J., Wang Z. (2019). Soluble ST2 and CD163 as potential biomarkers to differentiate primary hemophagocytic lymphohistiocytosis from macrophage activation syndrome. Mediterr. J. Hematol. Infect. Dis..

[42] Maruyama J., Inokuma S. (2010). Cytokine profiles of macrophage activation syndrome associated with rheumatic diseases. J. Rheumatol..

[43] Giamarellos-Bourboulis E.J., Netea M.G., Rovina N., Akinosoglou K., Antoniadou A., Antonakos N., Damoraki G., Gkavogianni T., Adami M.E., Katsaounou P., Ntaganou M., Kyriakopoulou M., Dimopoulos G., Koutsodimitropoulos I., Velissaris D. (2020). Complex immune dysregulation in COVID-19 patients with severe respiratory failure. Cell Host Microbe.

[44] Shimizu M., Yokoyama T., Yamada K., Kaneda H., Wada H., Wada T., Toma T., Ohta K., Kasahara Y., Yachie A. (2010). Distinct cytokine profiles of systemic-onset juvenile idiopathic arthritis-associated macrophage activation syndrome with particular emphasis on the role of interleukin-18 in its pathogenesis. Rheumatology (Oxford).

[45] Yasin S., Fall N., Brown R.A., Henderlight M., Canna S.W., Girard-Guyonvarc'h C., Gabay C., Grom A.A., Schulert G.S. (2020). IL-18 as a biomarker linking systemic juvenile idiopathic arthritis and macrophage activation syndrome. Rheumatology (Oxford).

[46] Ravelli A., Grom A.A., Behrens E.M., Cron R.Q. (2012). Macrophage activation syndrome as part of systemic juvenile idiopathic arthritis: Diagnosis, genetics, pathophysiology and treatment. Genes Immun..

[47] Sonmez H.E., Demir S., Bilginer Y., Ozen S. (2018). Anakinra treatment in macrophage activation syndrome: A single center experience and systemic review of literature. Clin. Rheumatol..

[48] Toldo S., Mezzaroma E., O'Brien L., Marchetti C., Seropian I.M., Voelkel N.F., Van Tassell B.W., Dinarello C.A., Abbate A. (2014). Interleukin-18 mediates interleukin-1-induced cardiac dysfunction. Am. J. Physiol. Heart Circ. Physiol..

[49] Gabay C., Fautrel B., Rech J., Spertini F., Feist E., Kotter I., Hachulla E., Morel J., Schaeverbeke T., Hamidou M.A., Martin T., Hellmich B., Lamprecht P., Schulze-Koops H., Courvoisier D.S. (2018). Open-label, multicentre, dose-escalating phase II clinical trial on the safety and efficacy of tadekinig alfa (IL-18BP) in adult-onset Still's disease. Ann. Rheum. Dis..

[50] Yasin S., Solomon K., Canna S.W., Girard-Guyonvarc'h C., Gabay C., Schiffrin E., Sleight A., Grom A.A., Schulert G.S. (2020). IL-18 as therapeutic target in a patient with resistant systemic juvenile idiopathic arthritis and recurrent macrophage activation syndrome. Rheumatology (Oxford).

[51] Li S., Neff C.P., Barber K., Hong J., Luo Y., Azam T., Palmer B.E., Fujita M., Garlanda C., Mantovani A., Kim S., Dinarello C.A. (2015). Extracellular forms of IL-37 inhibit innate inflammation *in vitro* and *in vivo* but require the IL-1 family decoy receptor IL-1R8. Proc. Natl. Acad. Sci. U. S. A..

[52] Nold-Petry C.A., Lo C.Y., Rudloff I., Elgass K.D., Li S., Gantier M.P., Lotz-Havla A.S., Gersting S.W., Cho S.X., Lao J.C., Ellisdon A.M., Rotter B., Azam T., Mangan N.E., Rossello F.J. (2015). IL-37 requires the receptors IL-18Ralpha and IL-1R8 (SIGIRR) to carry out its multifaceted anti-inflammatory program upon innate signal transduction. Nat. Immunol..

[53] Bufler P., Azam T., Gamboni-Robertson F., Reznikov L.L., Kumar S., Dinarello C.A., Kim S.H. (2002). A complex of the IL-1 homologue IL-1F7b and IL-18-binding protein reduces IL-18 activity. Proc. Natl. Acad. Sci. U. S. A..

[54] Yasuda K., Nakanishi K., Tsutsui H. (2019). Interleukin-18 in health and disease. Int. J. Mol. Sci..

[55] Charrad R., Berraies A., Hamdi B., Ammar J., Hamzaoui K., Hamzaoui A. (2016). Anti-inflammatory activity of IL-37 in asthmatic children: Correlation with inflammatory cytokines TNF-alpha, IL-beta, IL-6 and IL-17A. Immunobiology.

[56] Bouali E., Kaabachi W., Hamzaoui A., Hamzaoui K. (2015). Interleukin-37 expression is decreased in Behcet's disease and is associated with inflammation. Immunol. Lett..

[57] Jiang J.F., Xiao S.S., Xue M. (2018). Decreased expression of interleukin-37 in the ectopic and eutopic endometria of patients with adenomyosis. Gynecol. Endocrinol..

[58] Banda N.K., Vondracek A., Kraus D., Dinarello C.A., Kim S.H., Bendele A., Senaldi G., Arend W.P. (2003). Mechanisms of inhibition of collagen-induced arthritis by murine IL-18 binding protein. J. Immunol..

[59] Lewis E.C., Dinarello C.A. (2006). Responses of IL-18- and IL-18 receptor-deficient pancreatic islets with convergence of positive and negative signals for the IL-18 receptor. Proc. Natl. Acad. Sci. U. S. A..

[60] Nold-Petry C.A., Nold M.F., Nielsen J.W., Bustamante A., Zepp J.A., Storm K.A., Hong J.W., Kim S.H., Dinarello C.A. (2009). Increased cytokine production in interleukin-18 receptor alpha-deficient cells is associated with dysregulation of suppressors of cytokine signaling. J. Biol. Chem..

[61] Hojen J.F., Kristensen M.L.V., McKee A.S., Wade M.T., Azam T., Lunding L.P., de Graaf D.M., Swartzwelter B.J., Wegmann M., Tolstrup M., Beckman K., Fujita M., Fischer S., Dinarello C.A. (2019). IL-1R3 blockade broadly attenuates the functions of six members of the IL-1 family, revealing their contribution to models of disease. Nat. Immunol..

[62] Boraschi D., Tagliabue A. (2013). The interleukin-1 receptor family. Semin. Immunol..

[63] Dinarello C.A., Fantuzzi G. (2003). Interleukin-18 and host defense against infection. J. Infect. Dis..

[64] Wesche H., Korherr C., Kracht M., Falk W., Resch K., Martin M.U. (1997). The interleukin-1 receptor accessory protein (IL-1RAcP) is essential for IL-1-induced activation of interleukin-1 receptor-associated kinase (IRAK) and stress-activated protein kinases (SAP kinases). J. Biol. Chem..

[65] Huang J., Gao X., Li S., Cao Z. (1997). Recruitment of IRAK to the interleukin 1 receptor complex requires interleukin 1 receptor accessory protein. Proc. Natl. Acad. Sci. U. S. A..

[66] Kanakaraj P., Ngo K., Wu Y., Angulo A., Ghazal P., Harris C.A., Siekierka J.J., Peterson P.A., Fung-Leung W.P. (1999). Defective interleukin (IL)-18-mediated natural killer and T helper cell type 1 responses in IL-1 receptor-associated kinase (IRAK)-deficient mice. J. Exp. Med..

[67] Hezareh M., Hessell A.J., Jensen R.C., van de Winkel J.G., Parren P.W. (2001). Effector function activities of a panel of mutants of a broadly neutralizing antibody against human immunodeficiency virus type 1. J. Virol..

[68] Lee J.K., Kim S.H., Lewis E.C., Azam T., Reznikov L.L., Dinarello C.A. (2004). Differences in signaling pathways by IL-1beta and IL-18. Proc. Natl. Acad. Sci. U. S. A..

[69] Kim S.H., Reznikov L.L., Stuyt R.J., Selzman C.H., Fantuzzi G., Hoshino T., Young H.A., Dinarello C.A. (2001). Functional reconstitution and regulation of IL-18 activity by the IL- 18R beta chain. J. Immunol..

[70] Yoshimoto T., Takeda K., Tanaka T., Ohkusu K., Kashiwamura S., Okamura H., Akira S., Nakanishi K. (1998). IL-12 up-regulates IL-18 receptor expression on T cells, Th1 cells, and B cells: Synergism with IL-18 for IFN-gamma production. J. Immunol..

[71] Puren A.J., Fantuzzi G., Dinarello C.A. (1999). Gene expression, synthesis and secretion of IL-1b and IL-18 are differentially regulated in human blood mononuclear cells and mouse spleen cells. Proc. Natl. Acad. Sci. U. S. A..

[72] Puren A.J., Razeghi P., Fantuzzi G., Dinarello C.A. (1998). Interleukin-18 enhances lipopolysaccharide-induced interferon-gamma production in human whole blood cultures. J. Infect. Dis..

[73] Granowitz E.V., Vannier E., Poutsiaka D.D., Dinarello C.A. (1992). Effect of interleukin-1 (IL-1) blockade on cytokine synthesis: II. IL-1 receptor antagonist inhibits lipopolysaccharide-induced cytokine synthesis by human monocytes. Blood.

[74] Toth A., Csonka K., Jacobs C., Vagvolgyi C., Nosanchuk J.D., Netea M.G., Gacser A. (2013). Candida albicans and Candida parapsilosis induce different T-cell responses in human peripheral blood mononuclear cells. J. Infect. Dis..

[75] Netea M.G., Stuyt R.J., Kim S.H., Van der Meer J.W., Kullberg B.J., Dinarello C.A. (2002). The role of endogenous interleukin (IL)-18, IL-12, IL-1beta, and tumor necrosis factor-alpha in the production of interferon-gamma induced by Candida albicans in human whole-blood cultures. J. Infect. Dis..

[76] Nold M.F., Nold-Petry C.A., Zepp J.A., Palmer B.E., Bufler P., Dinarello C.A. (2010). IL-37 is a fundamental inhibitor of innate immunity. Nat. Immunol..

[77] van den Munckhof I.C.L., Kiki Schraa R.t.H., Stienstra R., de Graaf J., Riksen N.P., Joosten L.A.B., Netea M.G., Rutten J.H.W. (2019). IL-18 binding protein: A novel biomarker in obesity-related atherosclerosis that modulates lipoprotein metabolism. Atherosclerosis.

[78] Liu S., Miersch S., Li P., Bai B., Liu C., Qin W., Su J., Huang H., Pan J., Sidhu S.S., Wu D. (2020). A synthetic human antibody antagonizes IL-18Rbeta signaling through an allosteric mechanism. J. Mol. Biol..

[79] Ghezzi P., Dinarello C.A. (1988). IL-1 induces IL-1. III. Specific inhibition of IL-1 production by IFN-g. J. Immunol..

[80] Puren A.J., Fantuzzi G., Gu Y., Su M.S.-S., Dinarello C.A. (1998). Interleukin-18 (IFN-g-inducing factor) induces IL-1b and IL-8 via TNFa production from non-CD14+ human blood mononuclear cells. J. Clin. Invest.

[81] Dai S.M., Matsuno H., Nakamura H., Nishioka K., Yudoh K. (2004). Interleukin-18 enhances monocyte tumor necrosis factor alpha and interleukin-1beta production induced by direct contact with T lymphocytes: Implications in rheumatoid arthritis. Arthritis Rheum..

[82] Netea M.G., Kullberg B.J., Verschueren I., Van Der Meer J.W. (2000). Interleukin-18 induces production of proinflammatory cytokines in mice: No intermediate role for the cytokines of the tumor necrosis factor family and interleukin-1beta. Eur. J. Immunol..

[83] Stuyt R.J., Kim S.H., Reznikov L.L., Fantuzzi G., Novick D., Rubinstein M., Kullberg B.J., van der Meer J.W., Dinarello C.A., Netea M.G. (2003). Regulation of Staphylococcus epidermidis-induced IFN-gamma in whole human blood: The role of endogenous IL-18, IL-12, IL-1, and TNF. Cytokine.

[84] Manigold T., Bocker U., Traber P., Dong-Si T., Kurimoto M., Hanck C., Singer M.V., Rossol S. (2000). Lipopolysaccharide/endotoxin induces IL-18 via CD14 in human peripheral blood mononuclear cells *in vitro*. Cytokine.

[85] Cheng S.C., Joosten L.A., Kullberg B.J., Netea M.G. (2012). Interplay between Candida albicans and the mammalian innate host defense. Infect. Immun..

[86] Gauglitz G.G., Callenberg H., Weindl G., Korting H.C. (2012). Host defence against Candida albicans and the role of pattern-recognition receptors. Acta Derm. Venereol..

[87] Huang C., Wang Y., Li X., Ren L., Zhao J., Hu Y., Zhang L., Fan G., Xu J., Gu X., Cheng Z., Yu T., Xia J., Wei Y., Wu W. (2020). Clinical features of patients infected with 2019 novel coronavirus in Wuhan, China. Lancet.

[88] Chi Y., Ge Y., Wu B., Zhang W., Wu T., Wen T., Liu J., Guo X., Huang C., Jiao Y., Zhu F., Zhu B., Cui L. (2020). Serum cytokine and chemokine profile in relation to the severity of coronavirus disease 2019 in China. J. Infect. Dis..

[89] Lucas C., Wong P., Klein J., Castro T.B.R., Silva J., Sundaram M., Ellingson M.K., Mao T., Oh J.E., Israelow B., Takahashi T., Tokuyama M., Lu P., Venkataraman A., Park A. (2020). Longitudinal analyses reveal immunological misfiring in severe COVID-19. Nature.

[90] McGonagle D., Sharif K., O'Regan A., Bridgewood C. (2020). The role of cytokines including interleukin-6 in COVID-19 induced pneumonia and macrophage activation syndrome-like disease. Autoimmun. Rev..

[91] Soy M., Keser G., Atagunduz P., Tabak F., Atagunduz I., Kayhan S. (2020). Cytokine storm in COVID-19: Pathogenesis and overview of anti-inflammatory agents used in treatment. Clin. Rheumatol..

[92] Otsuka R., Seino K.I. (2020). Macrophage activation syndrome and COVID-19. Inflamm. Regen..

[93] Canna S.W., Behrens E.M. (2012). Making sense of the cytokine storm: A conceptual framework for understanding, diagnosing, and treating hemophagocytic syndromes. Pediatr. Clin. North Am..

[94] Huang K.J., Su I.J., Theron M., Wu Y.C., Lai S.K., Liu C.C., Lei H.Y. (2005). An interferon-gamma-related cytokine storm in SARS patients. J. Med. Virol..

[95] Vecchie A., Bonaventura A., Toldo S., Dagna L., Dinarello C.A., Abbate A. (2021). IL-18 and infections: Is there a role for targeted therapies?. J. Cell Physiol..

[96] Wen W., Su W., Tang H., Le W., Zhang X., Zheng Y., Liu X., Xie L., Li J., Ye J., Dong L., Cui X., Miao Y., Wang D., Dong J. (2020). Immune cell profiling of COVID-19 patients in the recovery stage by single-cell sequencing. Cell Discov..

[97] Eisenmesser E.Z., Gottschlich A., Redzic J.S., Paukovich N., Nix J.C., Azam T., Zhang L., Zhao R., Kieft J.S., The E., Meng X., Dinarello C.A. (2019). Interleukin-37 monomer is the active form for reducing innate immunity. Proc. Natl. Acad. Sci. U. S. A..

[98] Leoni F., Fossati G., Lewis E.C., Lee J.K., Porro G., Pagani P., Modena D., Moras M.L., Pozzi P., Reznikov L.L., Siegmund B., Fantuzzi G., Dinarello C.A., Mascagni P. (2005). The histone deacetylase inhibitor ITF2357 reduces production of pro-inflammatory cytokines *in vitro* and systemic inflammation *in vivo*. Mol. Med..

[99] Li S., Fossati G., Marchetti C., Modena D., Pozzi P., Reznikov L.L., Moras M.L., Azam T., Abbate A., Mascagni P., Dinarello C.A. (2015). Specific inhibition of histone deacetylase 8 reduces gene expression and production of proinflammatory cytokines *in vitro* and *in vivo*. J. Biol. Chem..

[100] van de Veerdonk F.L., Joosten L.A., Devesa I., Mora-Montes H.M., Kanneganti T.D., Dinarello C.A., van der Meer J.W., Gow N.A., Kullberg B.J., Netea M.G. (2009). Bypassing pathogen-induced inflammasome activation for the regulation of interleukin-1beta production by the fungal pathogen Candida albicans. J. Infect. Dis..

[101] van de Veerdonk F.L., Marijnissen R.J., Kullberg B.J., Koenen H.J., Cheng S.C., Joosten I., van den Berg W.B., Williams D.L., van der Meer J.W., Joosten L.A., Netea M.G. (2009). The macrophage mannose receptor induces IL-17 in response to Candida albicans. Cell Host Microbe.

