# Supplementary material for: A novel anti-human IL-1R7 antibody reduces IL-18-mediated inflammatory signaling
Source: J Biol Chem. 2021 Apr 3;296:100630. doi: 10.1016/j.jbc.2021.100630 (PMC8018910; doi:10.1016/j.jbc.2021.100630)
Supplement: Figures S1 to S6 [file mmc1.pdf]

Supporting Information

A novel anti-human IL-1R7 antibody reduces IL-18 mediated inflammatory signaling

Suzhao Li[1,#*], Liqiong Jiang[1,2#], Karsten Beckmann[3,7,#], Jesper Falkesgaard Højen[4], Ulrich Pessara[3], Nicholas E. Powers[1], Dennis M. de Graaf[1], Tania Azam[1], Jared Lindenberger[5], Elan Z. Eisenmesser[6], Stephan Fischer[3], and Charles A. Dinarello[1,*]

[1]Department of Medicine, University of Colorado Denver Anschutz Medical Campus, Aurora, CO 80045, USA
[2]Shenzhen Eye Hospital, No.18 Zetian Road, Futian District, Shenzhen, Guangdong, China 518040
[3]MAB Discovery GmbH, Neuried, Germany
[4]Department of Infectious Diseases, Aarhus University Hospital, 8200 Aarhus N, Denmark
[5]Biophysics Core facility, University of Colorado Denver Anschutz Medical Campus, Aurora, CO, USA
[6]Department of Biochemistry and Molecular Genetics, University of Colorado Denver Anschutz Medical Campus, Aurora, CO 80045, USA
[7]Present address: BioNTech SE, An der Goldgrube 12, 55131 Mainz, Germany

[#]These authors contributed equally to this work.
*To whom correspondence should be addressed: Suzhao Li and Charles A. Dinarello, Department of Medicine, University of Colorado Denver Anschutz Medical Campus, Aurora, Colorado 80045, USA,
Tel: 303-724-4920, Fax: 303 724-6178,
Email: suzhao.li@cuanschutz.edu, cdinare333@aol.com

Running title: Anti-IL-1R7 suppresses IL-18 pathway

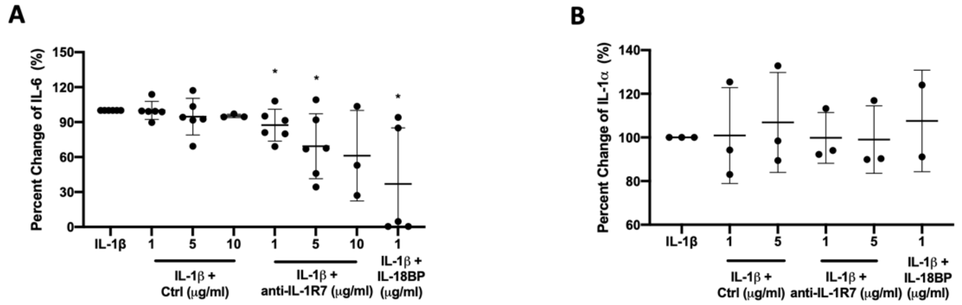

Supplemental Figure 1. Effect of anti-IL-1R7 on IL-1β-induced IL-6 and intracellular IL-1α in human lung epithelial A549-hIL-1R7 cell cultures. Cells are pretreated with or without isotype control, anti-IL-1R7 or IL-18BP for at least 30 minutes before they were stimulated with 1ng/ml IL-1β for 24 hours. Mean ± SD Percent change of IL-1β-induced cytokine production in human A549-hIL-1R7 cells with various concentrations of anti-IL-1R7 or its isotype control or IL-18BP (n ≧3 except for IL-1β +IL-18BP induced IL-1α where n=2). *P < 0.05, compared with IL-1β alone.

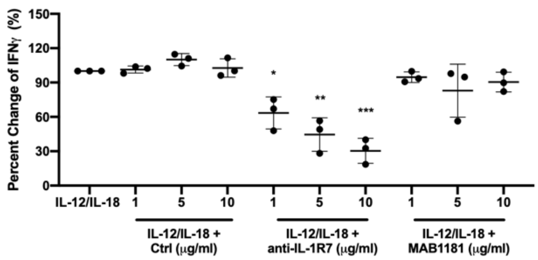

Supplemental Figure 2. The effect of anti-IL-1R7 in comparison to the reference antibody MAB1181 on the IL-12/IL-18-induced IFNγ in human PBMC cultures. Cells are pretreated with or without Ctrl, anti-IL-1R7 or the reference antibody MAB1181 for at least 30 minutes before they were stimulated with 2ng IL-12+20ng/ml IL-18 for 24 hours. Mean ± SD Percent change of IL-12/IL-18-induced IFNγ production in PBMCs with various concentrations of anti-IL-1R7 or its isotype control or the reference antibody MAB1181 (n=3 for all conditions). ***P < 0.001, **P < 0.01, *P < 0.05 compared with IL-12/IL-18 alone.

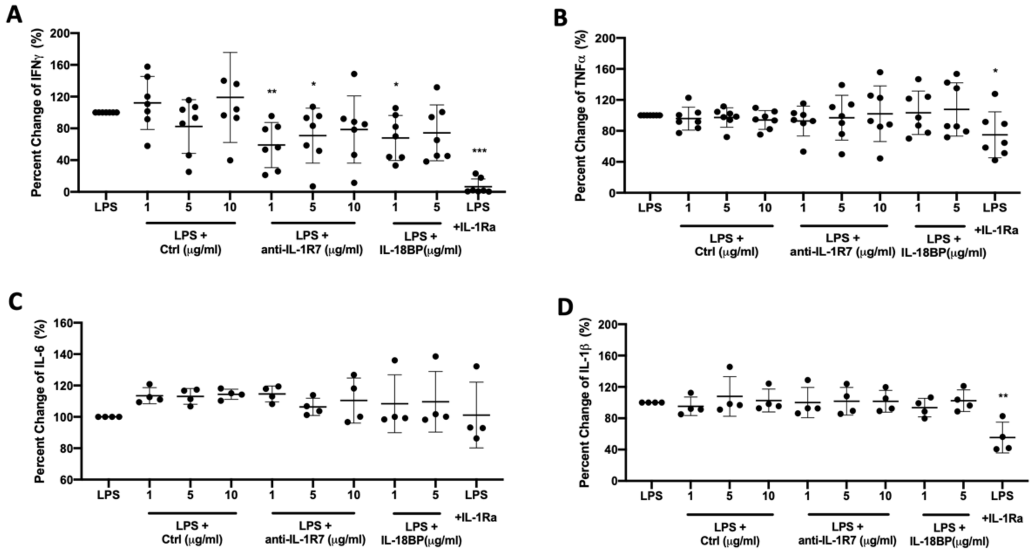

Supplemental Figure 3. The effect of anti-IL-1R7 antibody on 3-day LPS-induced cytokine production in human PBMC culture. Cells are pretreated with or without Ctrl, anti-IL-1R7 or IL-18BP or IL-1Ra for at least 30 minutes before they were stimulated with 10ng/ml LPS for 3 days. **(A)** Effect of anti-IL-1R7 on LPS-induced IFNγ in PBMC culture. **(B)** Effect of anti-IL-1R7 on LPS-induced TNFα in PBMC culture. **(C)** Effect of anti-IL-1R7 on LPS-induced IL-6 in PBMC culture. **(D)** Effect of anti-IL-1R7 on LPS-induced IL-1β in PBMC culture. Mean ± SD Percent change of LPS-induced cytokine production in PBMCs with various concentrations of anti-IL-1R7 or its isotype control or IL-18BP or IL-1Ra (n ≧4 for all conditions). ***P < 0.001, **P < 0.01, and *P < 0.05, compared with LPS alone.

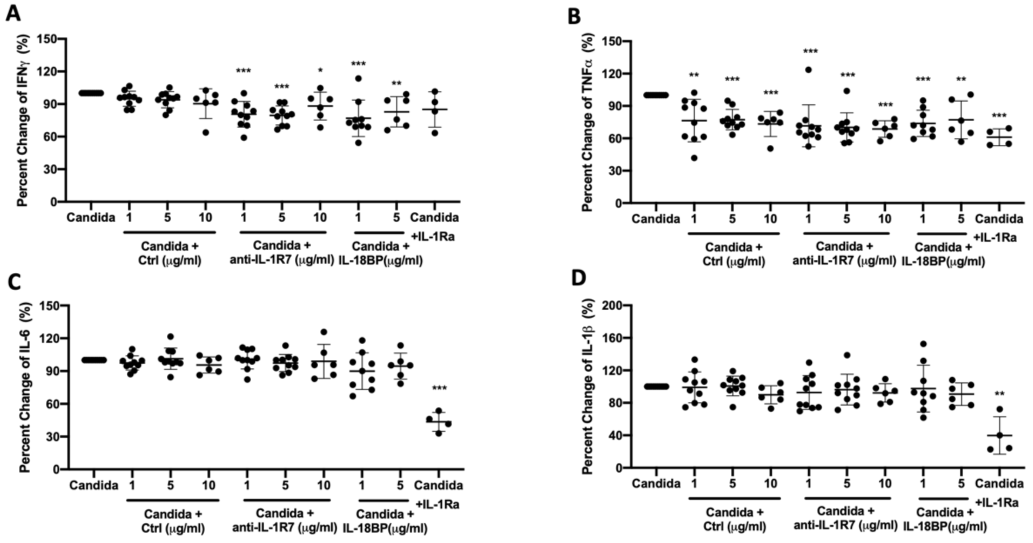

Supplemental Figure 4. The effect of anti-IL-1R7 antibody on *Candida*-induced cytokine production in human PBMC culture. Cells were pretreated with or without isotype control, anti-IL-1R7 or IL-18BP or IL-1Ra for at least 30 minutes before they were stimulated with 1:200 *Candida* for 3-5 days. **(A)** Effect of anti-IL-1R7 on *Candida*-induced IFNγ in PBMC culture. **(B)** Effect of anti-IL-1R7 on *Candida*-induced TNFα in PBMC culture. **(C)** Effect of anti-IL-1R7 on *Candida*-induced IL-6 in PBMC culture. **(D)** Effect of anti-IL-1R7 on *Candida*-induced IL-1β in PBMC culture. Mean ± SD Percent change of *Candida*-induced cytokine production in PBMCs with various concentrations of anti-IL-1R7 or its isotype control or IL-18BP or IL-1Ra (n ≧4 for all conditions). ***$P < 0.001$, **$P < 0.01$, and *$P < 0.05$, compared with *Candida* alone.

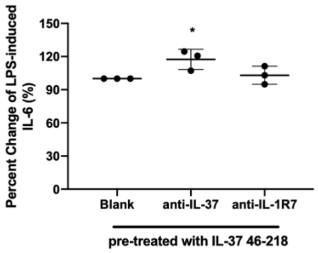

Supplemental Figure 5. The effect of anti-IL-1R7 in comparison to the monoclonal anti-IL-37 antibody on IL-37-regulated IL-6 production in LPS-stimulated PBMC. Cells were pretreated for 1 hour with Blank (RPMI medium premixed with 1ng/ml recombinant IL-37 46-218), or anti-IL-37 premixed with recombinant IL-37 46-218, or anti-IL-1R7 premixed with recombinant IL-37 46-218 before they were stimulated with 10ng/ml LPS for 24 hours. Mean ± SD Percent change of LPS-induced IL-6 production in PBMCs in the presence of 1ng/ml recombinant IL-37 pretreated with or without 1μg/ml anti-IL-1R7 or anti-IL-37 monoclonal antibodies (n=3 for all conditions). *$P < 0.05$ compared with Blank (IL-37 pretreatment alone).

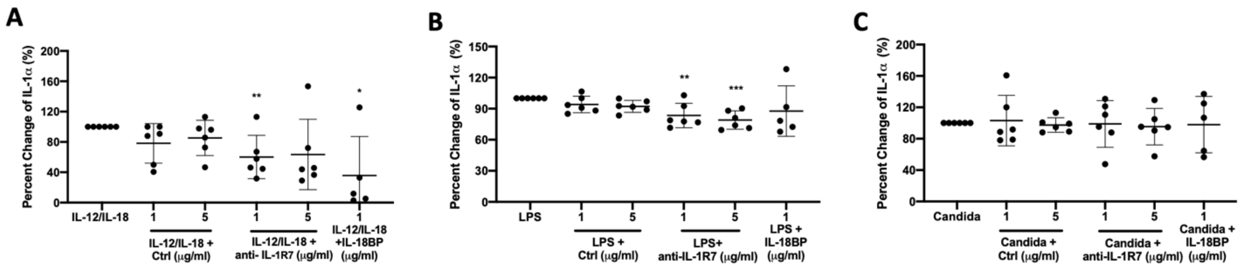

Supplemental Figure 6. Effect of anti-IL-1R7 on various stimuli-induced intracellular IL-1α production in PBMC cultures. Cells were pretreated with or without isotype control, anti-IL-1R7 or IL-18BP for at least 30 minutes before they were stimulated with **(A)** 2ng/ml IL-12+20ng/ml IL-18 or **(B)** 10ng/ml LPS or **(C)** 1:200 *Candida* for 1-5 days as indicated in *Experimental Procedures*. Mean ± SD Percent change of the corresponding stimulus-induced intracellular IL-1α production in PBMC with various concentrations of anti-IL-1R7 or its isotype control or IL-18BP (n ≧5). ***P < 0.001, **P < 0.01, and *P < 0.05, compared with the corresponding stimulus alone.
